# REAC Reparative Treatment: A Promising Therapeutic Option for Alcoholic Cirrhosis of the Liver

**DOI:** 10.3390/jpm13121698

**Published:** 2023-12-10

**Authors:** Lizomar de Jesus Maués Pereira, José Alfredo Coelho Pereira, Vania Fontani, Salvatore Rinaldi

**Affiliations:** 1 Faculty of Medicine, Federal University of Pará, Belem 66000-001, Brazil; 2 Research Department, Rinaldi Fontani Foundation, 50144 Florence, Italy; 3 Department of Reparative and Regenerative Medicine, Rinaldi Fontani Institute, 50144 Florence, Italy; 4 Department of Adaptive Neuro Psycho Physio Pathology and Neuro Psycho Physical Optimization, Rinaldi Fontani Institute, 50144 Florence, Italy

**Keywords:** alcoholic cirrhosis, epigenetics, reparative medicine, biomodulation, radio electric asymmetric conveyer, case report

## Abstract

Alcoholic liver disease (ALD) is a significant global health concern associated with excessive alcohol consumption. ALD encompasses various liver conditions with complex pathogenesis and progression influenced by environmental, genetic, and epigenetic factors. Alcoholic cirrhosis of the liver (ALC) is particularly prevalent among socially disadvantaged individuals, and current pharmacotherapy options provide limited treatment. This study aims to explore the potential benefits of radio electric asymmetric conveyer (REAC) technology and its tissue optimization reparative treatment (TO-RPR) in managing ALC. The liver possesses remarkable regenerative capabilities closely tied to its bioelectrical properties. REAC TO-RPR is a novel biotechnological therapeutic approach that aims to enhance and expedite reparative processes in injured tissues by restoring disrupted cellular endogenous bioelectric fields. This study seeks to optimize understanding of REAC TO-RPR’s impact on liver function and clinical outcomes in ALC patients. By investigating the mechanisms underlying liver’s reparative abilities and evaluating the efficacy of REAC TO-RPR, this research aims to address the urgent need for improved interventions in managing ALC. The findings hold potential for developing innovative treatment approaches, improving patient outcomes, and reducing the societal and individual burden associated with ALC.

## 1. Introduction

Alcoholic liver disease (ALD) is a global health concern attributed to excessive alcohol consumption. ALD encompasses a range of liver conditions, including steatosis, alcoholic steatohepatitis (ASH), fibrosis, cirrhosis, and hepatocellular carcinoma (HCC). The pathogenesis and progression of ALD are influenced by a complex interplay of environmental, genetic, and epigenetic factors [1,2,3,4].

Alcoholic cirrhosis of the liver (ALC) [5] is particularly prevalent among individuals belonging to disadvantaged social classes. Unfortunately, current pharmacotherapy options are unable to provide a permanent cure for ALC. The main therapeutic objective at present is to minimize the progression of the disease as much as possible (Table 1).

However, achieving abstinence from alcohol, the primary goal for patients with ALC, is often challenging due to the socioeconomic environments in which these individuals typically reside.

The liver possesses a remarkable capacity for regeneration, a process that is closely associated with its bioelectrical properties [6,7]. Previous research has investigated the endogenous bioelectrical activity (EBA) of the liver across various stages of disease [8]. EBA has been found to play a pivotal role in facilitating the recovery of liver tissue following injury [9].

In recent years, a novel biotechnological therapeutic approach as radio electric asymmetric conveyer (REAC) technology and its tissue optimization reparative-regenerative treatments (RRT), has emerged. These treatments have shown promise in enhancing and expediting the reparative-regenerative processes within tissues by restoring the cellular endogenous bioelectric fields that have been disrupted [10,11].

Expanding on this line of investigation, the present study aims to further explore the potential benefits of REAC RRT in the management of ALC. By harnessing the reparative power of the liver’s bioelectrical properties [6,7], it is hypothesized that REAC RRT may offer an effective intervention to facilitate tissue repair and potentially slow down the progression of ALC. 

This study aims to optimize our understanding of REAC RRT impact on liver function and clinical outcomes in patients with ALC. By investigating the mechanisms underlying the liver’s reparative abilities and evaluating the efficacy of REAC RRT, this research seeks to address the urgent requirement for improved interventions in managing ALC.

The findings from this case report hold the potential to facilitate the development of innovative treatment approaches, enhancing patient outcomes, and alleviating the societal and individual burden associated with ALC.

## 2. Case description

### 2.1. De-Identified Patient Information and Informed Consent

**Figure 1:**
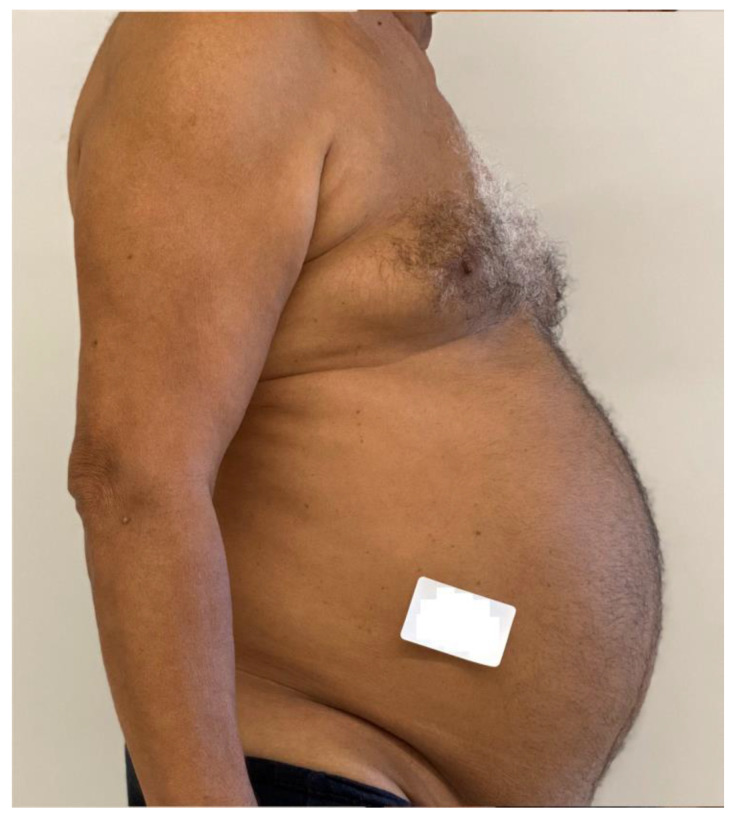
Profile image of the patient.

The patient was a 71-year-old man, weight: 76 kg, height: 1.52 m, BMI: 32.89 with a history of excessive alcohol consumption for more than 10 years, who was suffering from ALC. Written informed consent was obtained from the patient (Figure 1).

### 2.2. Relevant Physical Examination and Other Clinical Findings

The patient’s medical history, obtained through an anamnestic interview, revealed several significant comorbidities that pose challenges for managing ALC. These include insomnia, restless legs syndrome, diabetes, and difficult-to-control hypertension.

Insomnia [12,13] characterized by sleep disturbances, is a common comorbidity in patients with ALC and can significantly impact their quality of life. 

Restless legs syndrome [14,15], characterized by an irresistible urge to move the legs to relieve uncomfortable sensations, can further disrupt sleep and exacerbate the patient’s health challenges.

Diabetes adds complexity to the patient’s health profile [15,16]. This chronic metabolic disorder can affect multiple organ systems and lead to various complications. In the context of ALC, diabetes can further impair liver function and contribute to disease progression [16].

The patient’s difficult-to-control hypertension raises concerns regarding cardiovascular health. Hypertension, or high blood pressure, is a significant risk factor for heart disease, stroke, and other cardiovascular complications. In the case of ALC, hypertension can further strain the compromised liver, potentially worsening the disease progression [17,18].

The identification of these comorbidities during the anamnestic interview underscores the multifaceted and challenging nature of managing ALC in this patient.

For the aforementioned conditions, the patient had previously been prescribed the following medications and dosing regimens: Glifage XR 500 mg: two tablets after lunch and two tablets after dinner; Losartan 50 mg every 12 h; Hydrochlorothiazide 25 mg, one tablet in the morning; Pramipexole dihydrochloride: 0.75 mg; one tablet in the morning; Esomeprazole 40 mg once daily.

### 2.3. Relevant Past Interventions and Their Outcomes

The patient did not provide exact data on his alcohol abuse, but he still confirmed that his mental sobriety was altered almost daily, especially on weekends. Upon further inquiry, it was revealed that the patient, in addition to their prescribed medications for hypertension, was not undergoing any other therapeutic interventions or taking any additional medications to manage their health concerns.

## 3. Diagnostic Assessment

**Figure 2:**
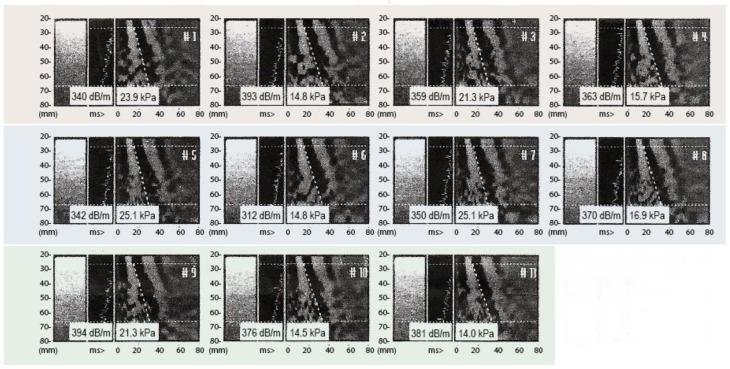
Fibro Scan examination pre REAC TO-RPR treatment. The figure shows the eleven correct acquisitions of the Fibro Scan examination necessary to elaborate the correct diagnosis.

In February, hepatic elastography was employed to assess the stage of ALC. The evaluation utilized the Fibro Scan^®^ device [19] (Echosens, Walhain, Belgium), a well-established method for measuring liver stiffness [20]. The elastography examination results revealed notable structural alterations in the liver, with an average stiffness measurement of 21.3 kPa (ranging from 14 to 25.7 kPa) and an interquartile range (IQR) to median ratio of 45.5% [21]. This stiffness value corresponds to the advanced stage of ALC (F4) on the widely recognized METAVIR scale [22] (Figure 2 and Table 2).

Hepatic elastography, a non-invasive imaging technique, has proven to be a valuable tool in assessing liver fibrosis and ALC [20]. By utilizing shear wave technology, elastography quantifies the stiffness of liver tissue, which serves as an indicator of fibrosis severity. The Fibro Scan^®^ device employs a low-frequency ultrasound probe to generate shear waves that propagate through the liver. The velocity of these waves is directly related to tissue stiffness, providing a quantitative measurement of liver fibrosis.

The obtained liver stiffness measurement of 21.3 kPa falls within the range previously documented for advanced ALC. It is crucial to note that the stiffness values measured by hepatic elastography exhibit a positive correlation with the degree of fibrosis progression. As such, higher stiffness values are indicative of more extensive fibrotic changes within the liver.

The METAVIR scale [22], widely adopted for the histopathological assessment of liver fibrosis, classifies ALC into different stages based on the degree of fibrotic alterations. The F4 stage represents the most advanced form of ALC, characterized by the presence of extensive fibrosis and architectural distortion within the liver parenchyma.

Figure 2 and Table 2 illustrate the obtained elastography results, highlighting the structural alterations observed in the liver and confirming the diagnosis of ALC (F4) according to the METAVIR scale. These visual representations further support the clinical findings and contribute to the comprehensive characterization of the patient’s liver disease status.

## 4. Therapeutic Intervention

To address the hepatic structural alterations observed in the patient, a therapeutic intervention involving an 18-session cycle of REAC TO-RPR neurobiological modulation [10,23] was recommended, which aims to facilitate reparative processes in both superficial and deep tissues.

Each REAC TO-RPR treatment session consisted of a standardized 30 min procedure. The treatment parameters were preprogrammed and could not be modified by the operator. During the sessions, the patient’s skin in the hepatic area was contacted with an asymmetric conveyer probe (ACP) connected to the REAC BENE model 110 device (ASMED Srl, in Florence, Italy). 

The REAC TO-RPR cycle was administered over a period of 6 weeks, with sessions conducted three times per week. This treatment regimen aimed to provide consistent and regular improvement to the hepatic area, promoting reparative processes and potentially mitigating the observed structural alterations in the liver.

By employing preprogrammed treatment parameters and ensuring standardized contact with the hepatic area using the ACP, this intervention aims to provide targeted stimulation for therapeutic purposes.

Administering the REAC TO-RPR cycle over the course of 6 weeks and scheduling sessions three times per week allows for a comprehensive treatment plan and an adequate timeframe for potential therapeutic effects to occur. This treatment approach acknowledges the need for repeated treatments to optimize reparative processes in the liver and reflects the intention to address the hepatic structural alterations observed in the patient.

## 5. Follow-Up and Outcomes

Roughly six months following the conclusion of the REAC TO-RRT, the patient underwent a subsequent liver assessment. The patient’s parameters of weight, height, and BMI remained unchanged compared to the initial assessment, namely: weight: 76 kg, height: 1.52 cm, BMI: 32.89. Furthermore, the patient’s dietary habits, alcohol consumption, and prior pharmacological therapy remained unchanged.

Given that the Fibroscan device employed for the prior evaluation was undergoing technical maintenance and repairs, the follow-up examination utilized a hepatic elastography with a Logiq S8 device, ensuring comprehensive comparability with assessments previously conducted using the Fibroscan [20]. This second assessment aimed to verify whether the REAC TO-RPR administered, following the biological time required for the functional reorganization of the liver, resulted in positive changes in hepatic tissue compared to the initial condition.

The liver exhibited normal topography, shape, dimensions, contours, and surface. A homogeneous echotexture of the liver parenchyma was observed, showing signs of mild fatty infiltration but without any focal lesions. Elastography was conducted using the shear wave method [19,24] with the Logiq S8 device developed by GE Healthcare (Chalfont St. Giles, Buckinghamshire, UK).

**Figure 3:**
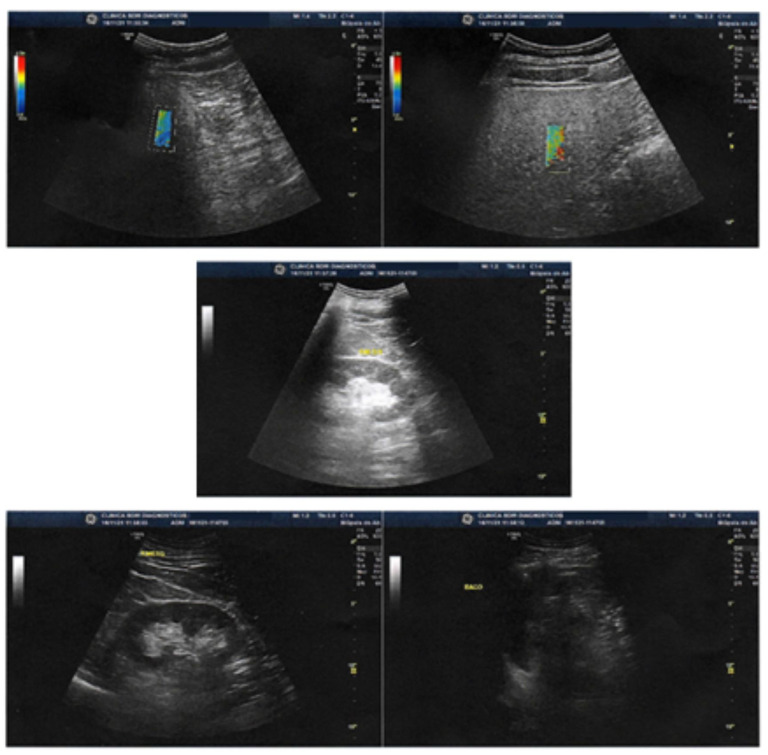
Images show liver with normal topography, shape, dimensions, contours and surface. Homogeneous liver parenchyma echotexture, with signs of mild fatty infiltration, without focal lesions. Elastography with the shear wave method (Logiq S8) showing mean stiffness (Kpa) of 5.70 corresponding to non-significant fibrosis (FO-F1) on the METAVIR scale.

**Figure 4:**
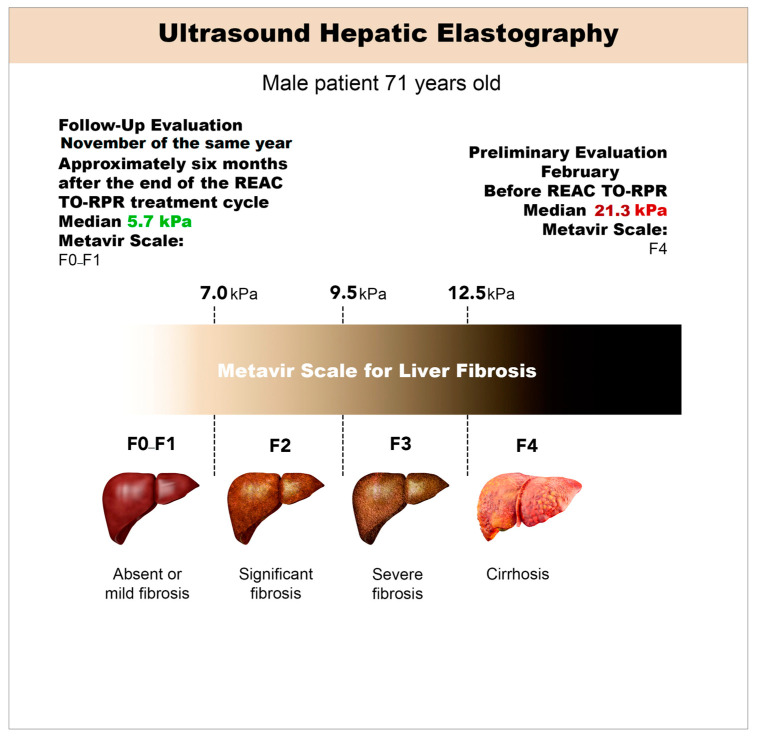
Synthetic representation of the evolution of the therapeutic response determined by the REAC TO-RPR treatment cycle.

The elastography results demonstrated a mean stiffness measurement of 5.70 kPa, which corresponds to non-significant fibrosis (F0–F1) on the METAVIR scale [22]. The stiffness range fell between 2.5 and 7 kPa, indicating minimal fibrotic changes within the liver (Figure 3 and Figure 4, Table 3). The IQR/median was 15.4%.

The observed normalization of liver topography, shape, and echotexture, along with the absence of focal lesions, suggests an improvement in liver health following the REAC RRT cycle. The reduction in liver stiffness measurements and the transition to a non-significant fibrosis stage (F0–F1) further support the potential beneficial effects of the intervention.

It is noteworthy that these positive outcomes were achieved despite the patient’s continued alcohol abuse during the treatment period. The findings suggest that REAC RRT treatment may have contributed to the improvement of liver fibrosis.

The utilization of the shear wave elastography technique with the Logiq S8 device allowed for accurate and quantitative assessment of liver stiffness, providing valuable insights into the patient’s liver health status. The accompanying figures (Figure 3 and Figure 4) and table (Table 3) visually depict the elastography results, reinforcing the observed improvements in liver structure and fibrosis severity.

These follow-up outcomes emphasize the potential of REAC TO-RPR treatment in promoting liver health and mitigating the adverse effects of ALC, even in the presence of ongoing alcohol abuse. Further research and long-term studies are warranted to validate these findings and elucidate the underlying mechanisms of TO-RPR treatment in liver reparation, regeneration and fibrosis mitigation.

## 6. Patient Perspective

Taking into account the notable outcomes observed following a single cycle of REAC TO-RPR treatment, the patient’s perspective in terms of prognosis appears promising. It is crucial, however, to emphasize to the patient the importance of comprehending the potential adverse implications of ongoing alcohol abuse on their overall health. The patient should be made aware that limiting or completely abstaining from alcohol consumption plays a vital role in preserving their health and well-being. By adhering to this recommendation, the patient can optimize the positive effects achieved through the REAC TO-RPR treatment and enhance their long-term prognosis.

The present case report provides compelling evidence supporting the potential effectiveness of REAC TO-RPR manipulation of endogenous bioelectrical activity (EBA) in reversing hepatic fibrosis in individuals diagnosed with ALC of the liver. Remarkably, the patient in this study continued to engage in alcohol abuse throughout both the treatment and follow-up periods, highlighting the robustness of the REAC TO-RPR approach.

Despite ongoing alcohol abuse, the results demonstrated a significant and sustained improvement in hepatic fibrosis following the intervention, which persisted for more than 6 months post-treatment. These findings carry substantial clinical significance, considering the inherent challenges associated with managing patients suffering from chronic liver disease and the limited efficacy of currently available therapeutic options.

The utilization of REAC TO-RPR for neurobiological modulation, specifically in manipulating the endogenous bioelectrical activity, presents a promising avenue for the treatment of liver fibrosis in individuals with ALC. This study’s findings underscore the critical need for the development of novel and effective therapeutic modalities tailored to address the specific challenges faced by this patient population. Moreover, they warrant further investigation and research to comprehensively understand the mechanisms underlying the observed positive outcomes and to refine and optimize the REAC TO-RPR approach.

## 7. Discussion

ALD is a prevalent chronic liver condition that presents considerable challenges in terms of diagnosis and treatment. Its impact on public health is substantial, leading to elevated rates of illness and death. To effectively address this urgent issue, it is imperative to explore innovative research avenues that can enhance our comprehension of ALD and its associated complications. One emerging area of investigation focuses on the role of epigenetic changes in the development and progression of various disease states, including the development and advancement of liver disorders, particularly hepatic fibrogenesis and ALC [1,2,4].

Notably, alcohol consumption has been found to have profound effects on liver epigenetics, playing a key role in the etiology of ALD.

Alcohol-induced epigenetic modifications within the liver involve a range of molecular processes. These include alterations in histone modifications, genetic modulation induced by microRNAs, changes in DNA methylation patterns, and alcohol-triggered cell signaling mechanisms that impact gene expression [1,2,3,4,25]. These epigenetic changes are instrumental in driving the pathogenesis of ALD, contributing to the molecular mechanisms underlying liver damage and disease progression [1,2,4].

In this study, we aimed to contribute to the understanding and treatment of ALC by utilizing REAC TO-RPR treatment. REAC technology is specifically designed to optimize the endogenous bioelectrical activity (EBA), which plays a crucial role in all cellular functions, including those associated with epigenetic modifications [25,26]. Prior research has provided compelling evidence showcasing the efficacy of distinct REAC treatment protocols in orchestrating epigenetic reprogramming across diverse cell lines [27].

Our study focused on investigating the potential of REAC TO-RPR treatment to induce functional reorganization in tissues affected by harmful agents and epigenetic conditions [10,23].

Although our findings are based on a single case report, they are consistent with the expected outcomes derived from previous studies [27].

By exploring the effects of REAC TO-RPR treatment in ALC, we aim to contribute to the growing body of knowledge in this field and pave the way for potential advancements in the treatment of this complex condition.

It is important to conduct additional studies to validate and refine our results, thereby enhancing our understanding and optimizing therapeutic approaches for ALC.

## Figures and Tables

**Table 1:** Current therapeutic intervention in ALC.

Therapeutic Intervention for ALC	Mechanism of Action	Benefits	Limitations
Nutritional Therapy	Provides essential nutrients to support liver function and prevent malnutrition.	Improves overall health and reduces the risk of complications.	May not be effective in advanced stages of cirrhosis.
Abstinence from Alcohol	Prevents further liver damage and allows for liver regeneration.	Essential for long-term survival and improvement of liver function.	May be difficult to achieve for individuals with alcohol dependence.
Diuretics	Reduce fluid accumulation in the abdomen (ascites) and legs (edema).	Improves quality of life and reduces symptoms such as abdominal discomfort and shortness of breath.	May cause electrolyte imbalances and dehydration.
Lactulose	Prevents and treats hepatic encephalopathy, a complication of cirrhosis that causes confusion and impaired mental function.	Improves neurologic function and reduces the risk of coma.	May cause diarrhea and abdominal discomfort.
Antibiotics	Prevent and treat bacterial infections, which are more common in individuals with cirrhosis.	Reduces the risk of serious infections that can worsen liver function.	Can contribute to antibiotic resistance.
Endoscopic Therapy	Treats complications of cirrhosis, such as esophageal variceal bleeding.	Reduces the risk of life-threatening bleeding.	May require repeated procedures.
Liver Transplantation	Replaces the diseased liver with a healthy one.	Cures cirrhosis and improves survival.	Requires a suitable donor liver and carries the risks of surgery and immunosuppression.

**Table 2:** Pre-treatment Fibroscan results.

Pre-Treatment Fibroscan Results											
E1	E2	E3	E4	E5	E6	E7	E8	E9	E10	E11	Median
23.9	14.8	21.3	25.7	25.1	14.8	25.1	16.9	21.3	14.5	14.0	21.3
Kpa	Kpa	Kpa	Kpa	Kpa	Kpa	Kpa	Kpa	Kpa	Kpa	Kpa	Kpa

**Table 3:** Post-treatment ultrasound liver elastography results.

Post-Treatment Ultrasound Liver Elastography Results												
E1	E2	E3	E4	E5	E6	E7	E8	E9	E10	E11	E12	Median
7.78	6.02	6.06	6.58	5.41	5.64	4.14	5.02	7.41	4.37	5.76	5.50	5.70
Kpa	Kpa	Kpa	Kpa	Kpa	Kpa	Kpa	Kpa	Kpa	Kpa	Kpa	Kpa	Kpa

## Data Availability

All data are included in the manuscript.
